# Sulfur metabolism-mediated fungal glutathione biosynthesis is essential for oxidative stress resistance and pathogenicity in the plant pathogenic fungus *Fusarium graminearum*

**DOI:** 10.1128/mbio.02401-23

**Published:** 2023-12-19

**Authors:** Jiyeun Park, Jae Woo Han, Nahyun Lee, Sieun Kim, Soyoung Choi, Hyun-Hee Lee, Jung-Eun Kim, Young-Su Seo, Gyung Ja Choi, Yin-Won Lee, Hun Kim, Hokyoung Son

**Affiliations:** 1 Department of Agricultural Biotechnology, Seoul National University, Seoul, South Korea; 2 Center for Eco-Friendly New Materials, Korea Research Institute of Chemical Technology, Daejeon, South Korea; 3 Department of Integrated Biological Science, Pusan National University, Busan, South Korea; 4 Research Institute of Climate Change and Agriculture, National Institute of Horticultural and Herbal Science, Jeju, South Korea; 5 Department of Medicinal Chemistry and Pharmacology, University of Science and Technology, Daejeon, South Korea; 6 Research Institute of Agriculture and Life Sciences, Seoul National University, Seoul, South Korea; Max Planck Institute for Terrestrial Microbiology, Marburg, Germany

**Keywords:** oxidative stress response, sulfur metabolism, glutathione, γ-glutamylcysteine, pathogenicity

## Abstract

The oxidative stress response is required for plant pathogens to endure host-derived oxidative stress during infection. Previously, we identified the eight transcription factors (TFs) involved in the oxidative stress response in the plant pathogenic fungus *Fusarium graminearum* and found that of these TFs, the deletion of *FgbZIP007* caused hypersensitivity to oxidative stress. However, the underlying mechanisms of Fgbzip007 are not fully understood. Based on chromatin immunoprecipitation followed by sequencing (ChIP-seq) analysis, we found the regulons of Fgbzip007, and further genetic studies demonstrated that Fgbzip007 is a key regulator for sulfur assimilation. The deletion strains of *FgbZIP007* and its regulons exhibited low level of glutathione biosynthesis, which led to characterize glutathione biosynthesis. Fgbzip007-mediated sulfur assimilation is required for glutathione biosynthesis, which is essential for oxidative stress resistance and pathogenicity in *F. graminearum*. Although the reduced resistance of glutathione-deficient mutants against oxidative stress was restored by overexpression of *FCA7*, encoding a core peroxidase, but not on pathogenicity, suggesting that glutathione in pathogenesis is independent of antioxidant properties. This study characterized the function of genes of glutathione biosynthesis, provides specific insight into how Fgbzip007 regulates pathogenesis in *F. graminearum*, and establishes a genetic framework for the molecular dissection of a TF Fgbzip007 with the integration of pathogen responses to oxidative stress.

**IMPORTANCE:**

*Fusarium graminearum* is a destructive fungal pathogen that causes Fusarium head blight (FHB) on a wide range of cereal crops. To control fungal diseases, it is essential to comprehend the pathogenic mechanisms that enable fungi to overcome host defenses during infection. Pathogens require an oxidative stress response to overcome host-derived oxidative stress. Here, we identify the underlying mechanisms of the Fgbzip007-mediated oxidative stress response in *F. graminearum*. ChIP-seq and subsequent genetic analyses revealed that the role of glutathione in pathogenesis is not dependent on antioxidant functions in *F. graminearum*. Altogether, this study establishes a comprehensive framework for the Fgbzip007 regulon on pathogenicity and oxidative stress responses, offering a new perspective on the role of glutathione in pathogenicity.

## INTRODUCTION

Fusarium head blight (FHB) is a destructive fungal disease that reduces the yield of cereal crops, leading to significant economic losses worldwide (1, 2). *Fusarium graminearum* is the primary causal agent of FHB (3), and this fungus produces mycotoxins such as trichothecenes and zearalenone. Mycotoxin contamination of the grains diminishes grain quality and poses a potential threat to human and animal health (4, 5). Several strategies, including crop breeding for disease resistance and chemical control, have been utilized for decades to manage the FHB (6, 7), but there are still limitations (8). Thus, it is necessary to understand the mechanisms of fungal development and pathogenesis in *F. graminearum* to develop new control strategies.

In plant-pathogen interactions, plants recognize the pathogen-associated molecular patterns (PAMP) and activate PAMP-triggered immunity (PTI) (9). Pathogens have weapons called “effectors,” which can suppress PTI, but plants also evolve to recognize the effectors *via* leucine-rich-repeat-containing receptors that activate effector-triggered immunity (ETI) (10). During PTI and ETI, the rapid production of reactive oxygen species (ROS), referred to as the oxidative burst, occurs in plant cells (11). Accumulation of ROS causes the direct killing of pathogens and induces hypersensitive reactions, which also prevents the spread of pathogens (12, 13). At the same time, the pathogens have efficient antioxidant mechanisms to protect themselves from the high concentration of ROS and invade successfully. The fungal antioxidant systems dealing with ROS derived from the host have been explored in plant pathogenic fungal species. It was found that several antioxidant systems, including superoxide dismutase and peroxidases, play a crucial role in pathogenicity, with increased expression during infection and in response to oxidative stress conditions (14–17).

Peroxidase, a major group of enzymatic antioxidants, participates in the oxidative stress response by reducing hydroperoxides (18), and in *F. graminearum*, the fungal peroxidases have been identified, and their role in oxidative stress resistance and pathogenicity were confirmed (19, 20). In our previous study, a total of 31 putative peroxidases were investigated, and Fca7, a catalase-peroxidase, was revealed as a key antioxidant enzyme that is required for pathogenicity (20). In addition, we screened a mutant library of transcription factors under oxidative stress conditions and identified eight transcription factors (TFs) playing a role in oxidative stress resistance: Zif1, Fgap1, Fgskn7, Fgzc086, Fgzc236, Fghome001, Fgc2h010, and Fgbzip007. Those TFs were collectively involved in the regulation of *FCA7* expression, and it was confirmed that the overexpression of *FCA7* restored the defects in most of the TF deletion mutants under oxidative stress conditions. However, the Δ*fgbzip007* mutant, which exhibited the highest susceptibility among those TF mutants, did not recover from its defects even with the overexpression of *FCA7*, and the underlying mechanisms of Fgbzip007 on oxidative stress response and pathogenicity remain unknown.

Given these previous results, we hypothesized that Fgbzip007 regulates different antioxidant system oxidative stress response mechanisms beyond the Fca7-mediated enzymatic antioxidant system and aimed to identify the underlying mechanisms of Fgbzip007 on oxidative stress response and pathogenicity. Here, we investigated the molecular mechanisms underlying pathogenesis in the plant pathogenic fungus *F. graminearum*, with particular emphasis on understanding the molecular basis of glutathione biosynthesis through Fgbzip007. An initial discovery that Fgbzip007 was required for oxidative stress resistance and pathogenicity in *F. graminearum* led to the discovery of Fgbzip007 regulons. Based on chromatin immunoprecipitation followed by high-throughput sequencing (ChIP-seq), we identified sulfur assimilation genes as Fgbzip007 regulons, which are necessary for glutathione biosynthesis. Our findings identify a novel molecular mechanism through which glutathione is essential for oxidative stress resistance and pathogenesis, which has led to the formulation of new hypotheses regarding the coordination of fungal pathogenesis and plant defense responses.

## RESULTS

### Identification and characterization of *FgbZIP007* in *F. graminearum*

**Fig 1:**
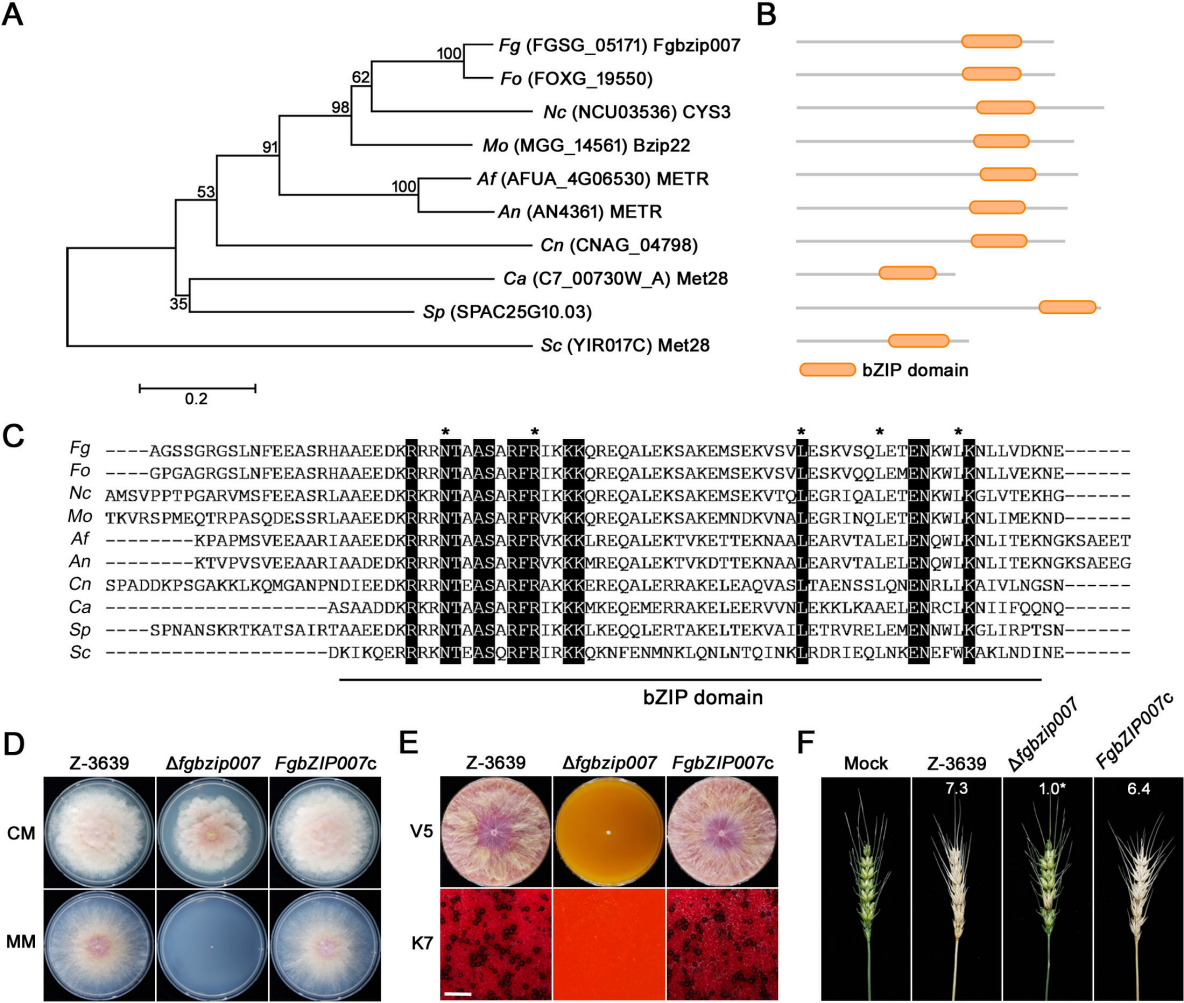
Identification and characterization of Fgbzip007. (**A**) Phylogenetic tree of Fgbzip007 orthologs in kingdom fungi. A phylogenetic tree was generated by the neighbor-joining method using the MEGA7 program. *Fg*, *Fusarium graminearum; Fo*, *F. oxysporum; Nc*, *Neurospora crassa; Mo*, *Magnaporthe oryzae; Af*, *Aspergillus fumigatus; An*, *A. nidulans; Cn*, *Cryptococcus neoformans; Ca*, *Candida albicans; Sp*, *Schizosaccharomyces pombe; Sc*, *Saccharomyces cerevisiae*. (**B**) Corresponding protein domain structures of Fgbzip007 orthologs. Protein domain information was obtained from the InterPro database. (**C**) Multiple sequence alignment of the conserved bZIP domains of Fgbzip007 orthologs. Amino acid sequences were aligned by the MUSCLE algorithm using the MEGA11 program. Asterisks indicate the conserved amino acid residues within the bZIP domain. White letters with black backgrounds indicate the amino acid residues conserved across all sequences. (**D**) Vegetative growth of each strain on complete medium (CM) and minimal medium (MM). Photographs of mycelial growth were taken 4 days (MM) and 5 days (CM) after inoculation. (**E**) Sexual reproduction of each strain on carrot agar. Photographs were taken 5 days after inoculation and 7 days after sexual induction. Scale bar = 1,000 µm. (**F**) Virulence on wheat heads. The center spikelet of each wheat head was injected with 10 µL of a conidial suspension. Photographs were captured 21 days after inoculation.

We identified Fgbzip007 homologs in several fungal species, and phylogenetic analysis indicated that Fgbzip007 is a homolog of CYS3/METR (21, 22) (Fig. 1A). BLASTp analysis revealed that Fgbzip007 was 40% identical to CYS3 of *Neurospora crassa* and 35% identical to METR of *Aspergillus nidulans*. A domain analysis using the InterPro database revealed that all Fgbzip007 homologs only contain a single basic leucine zipper (bZIP) domain, which was highly conserved in tested fungal species (Fig. 1B and C).

To characterize the function of *FgbZIP007* in *F. graminearum*, we generated *FgbZIP007* complemented strains and investigated the role of *FgbZIP007* in fungal development and pathogenicity. When fungal strains were grown on complete medium (CM) and minimal medium (MM), the radial growth of Δ*fgbzip007* deletion mutant was slightly reduced compared with the wild-type strain on CM but the growth defect was restored in the complemented strains (Fig. 1D). The deletion mutant was unable to grow on MM, and the growth impairment was also restored in the complemented strain (Fig. 1D). To investigate the sexual reproduction ability of the Δ*fgbzip007* mutant, each strain was inoculated on a carrot agar medium (CA). The wild-type and the complemented strains exhibited normal growth on CA and produced mature perithecia. By contrast, the Δ*fgbzip007* mutant showed a severe growth defect on CA, only forming white aerial mycelia on the surface. Subsequently, sexual reproduction did not occur in the deletion mutant (Fig. 1E).

To determine the pathogenicity of Δ*fgbzip007* mutants, the conidial suspension of each strain was inoculated on the middle spikelets of the flowering wheat heads. Spikelets inoculated with the wild-type and complemented strains turned light brown and desiccated, exhibiting typical symptoms of FHB. By contrast, the Δ*fgbzip007* mutant caused symptoms only in the inoculated spikelet and did not spread to uninoculated sites (Fig. 1F). These results suggest that Fgbzip007, a homolog of CYS3/METR, is essential for fungal development and virulence in *F. graminearum*.

### ChIP-seq revealed that Fgbzip007 is a key regulator of sulfur metabolism in *F. graminearum*

**Fig 2:**
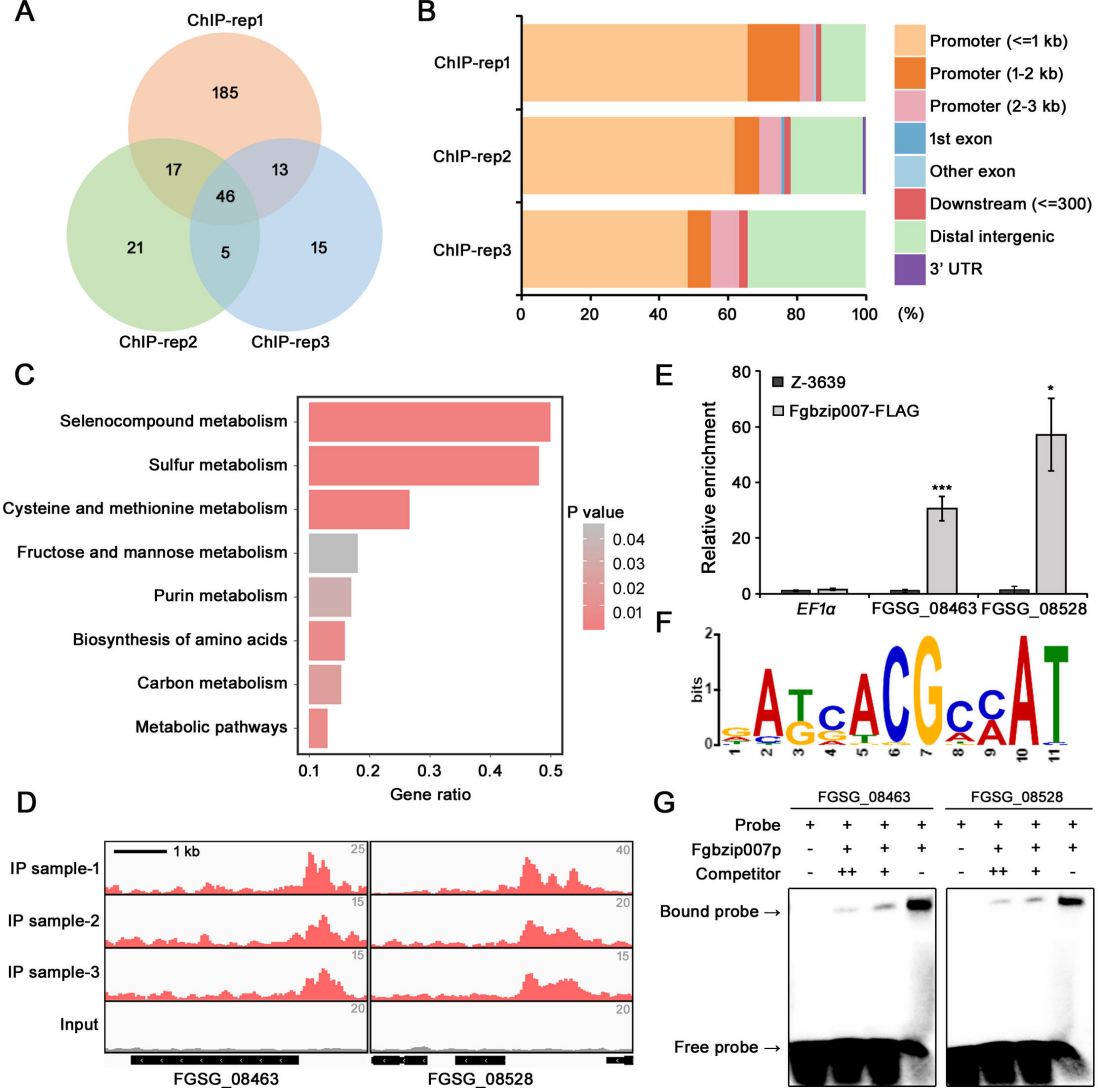
ChIP-seq analysis on Fgbzip007. (**A**) Venn diagram representing the number of peak-associated genes in each replicate. (**B**) Relative distribution of the peaks identified by ChIP-seq in each replicate. (**C**) KEGG enrichment analysis of the peak-associated genes. The y-axis represents the enriched pathway, and the x-axis represents the rich factor. (**D**) Visualization of the ChIP-seq peaks within two loci using an integrative genome viewer. (**E**) ChIP-qPCR analysis. *EF1α* was used as a negative control. Asterisks represent significant differences from the wild type (*P < 0.05; ***P < 0.001; *t*-test). (**F**) Fgbzip007 binding motif analyzed by MEME (*E*-value = 3.9e^−019^) (**G**) Electrophoretic mobility shift assay (EMSA). EMSA was performed using the probes within the binding motif identified in each gene. The “+” indicates the relevant protein and probes, and the “-” indicates their absence.

We performed ChIP-seq to identify the genes directly regulated by Fgbzip007 with optimized Fgbzip007 expression conditions. The transcript levels of *FgbZIP007* were induced twofold to threefold after transferring the mycelia to the sulfur-depleted condition; this result was consistent with a previous report on CYS3 of *N. crassa* (23) (Fig. S1A). Furthermore, western blot analysis showed that Fgbzip007 protein first appeared 2 h after sulfur limitation, and protein levels continued to increase until 8 h (Fig. S1B and C). Based on these results, we used the mycelia 4 h after transfer to sulfur-deficient conditions for ChIP analysis. We analyzed the immunoprecipitated DNA fragments of three independent ChIP experiments, and the reads obtained from sequencing were mapped to the *F. graminearum* PH-1 genome (24). In each replicate, a total of 261, 89, and 79 peaks were determined, and 46 peaks were shared by all replicates (Fig. 2A). More than 50% of peaks were located in the promoter region across all three replicates (Fig. 2B). To identify the molecular function of Fgbzip007 direct target genes, we performed enrichment analysis on 302 peak-associated genes and found that the genes were primarily enriched in “selenocompound metabolism” (fgr00450), “sulfur metabolism” (fgr00920), and “cysteine and methionine metabolism” (fgr00270) (Fig. 2C).

To validate the ChIP-seq results, we performed chromatin immunoprecipitation followed by quantitative PCR (ChIP-qPCR) on FGSG_08463 and FGSG_08528 which were enriched in sulfur metabolism categories. The visualization by Integrative Genomics Viewer (IGV) revealed that the peaks were predominantly enriched in the promoter regions of the two genes (Fig. 2D). ChIP-qPCR results showed significant enrichment of FGSG_08463 and FGSG_08528 in the immunoprecipitated Fgbzip007-FLAG sample; the enrichment levels of the FGSG_08463 and FGSG_08528 genes in the Fgbzip007-FLAG sample were approximately 28-fold and 41-fold higher than in the wild-type sample, respectively (Fig. 2E).

Based on Multiple EM for Motif Elicitation (MEME) analysis, a consensus sequence 5′-GAKCACGCMAT-3′ was identified in the genomic regions commonly detected in the call peaks of the three replicates (Fig. 2F). In addition, the result of electrophoretic mobility shift assay (EMSA) supported that the purified Fgbzip007 protein bound directly to the consensus sequence in the promoter regions of FGSG_08463 and FGSG_08528 (Fig. 2G).

### Fgbzip007 regulates the expression of sulfur assimilation-related genes in *F. graminearum*

**Fig 3:**
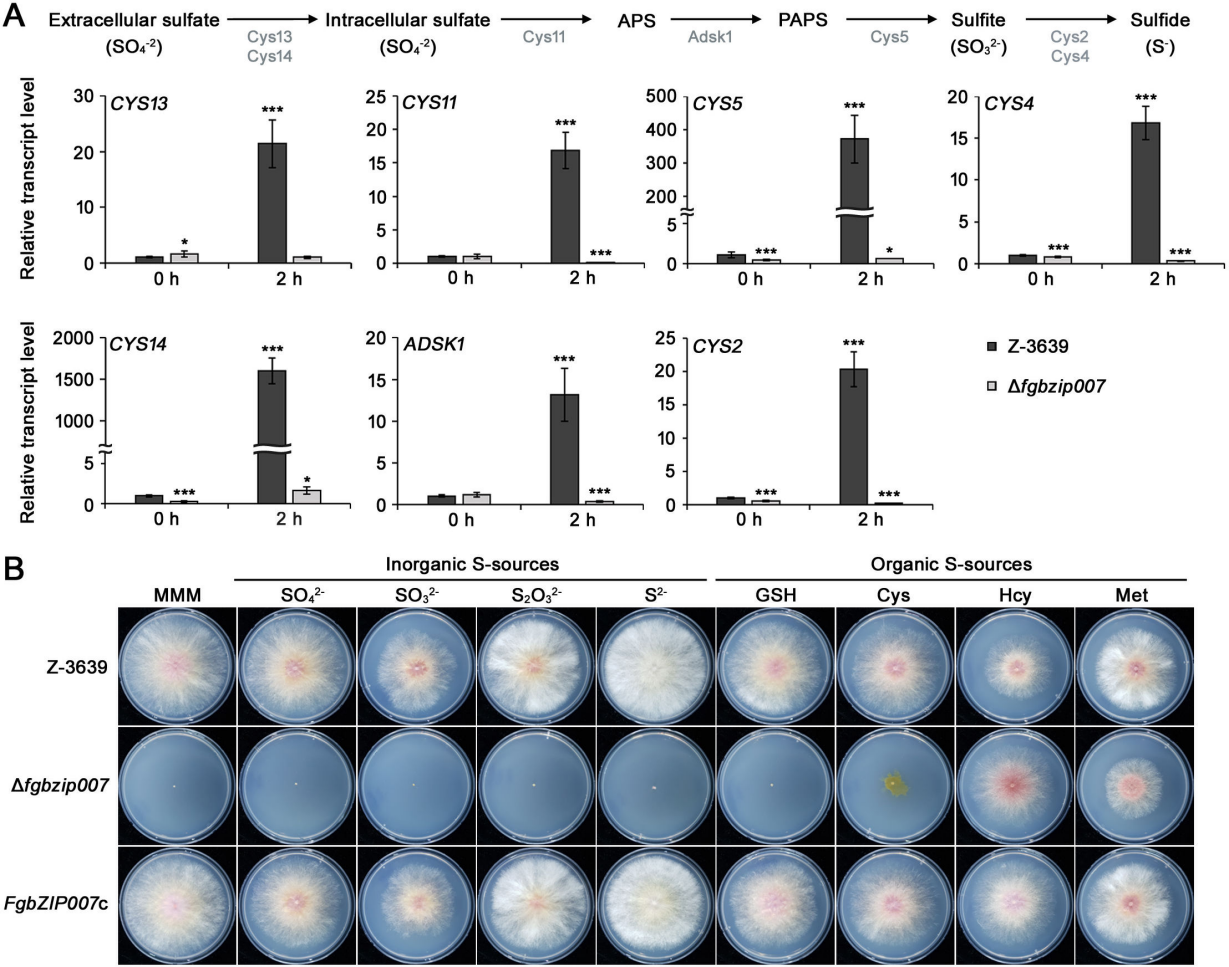
The role of Fgbzip007 in the regulation of sulfur assimilation. (**A**) Schematic of sulfur assimilation pathway in *F. graminearum* and expression profiles of sulfur assimilation pathway genes under sulfur-deprived conditions. The schematic of the sulfur assimilation pathway in *F. graminearum* is modified from the pathway in the previous study of *N. crassa* (25). APS, adenosine 5′-phosphosulfate; PAPS, 3′-phosphoadenosine-5′-phosphosulfate. The relative transcript levels of the genes encoding each enzyme are shown below the schematic pathway. Asterisks represent significant differences from the wild type (*P < 0.05; **P < 0.01; ***P < 0.001; *t*-test). (**B**) Vegetative growth of the wild-type, Δ*fgbzip007* and Fgbzip007c strains on modified minimal media (MMM) with various sulfur sources. GSH, glutathione; Cys, cysteine; Hcy, homocysteine; Met, methionine. Photographs were taken 4 days after inoculation.

Our results showing that Fgbzip007 interacts with FGSG_08463 and FGSG_08528 involved in sulfur metabolism led us to identify the genes encoding enzymes involved in the sulfur assimilation pathway in *F. graminearum*. Based on previous reports that identified genes for the sulfur assimilation pathway in *N. crassa* and *A. nidulans* (25, 26), we identified seven genes, including FGSG_08463 and FGSG_08528, in this study that encode sulfate permease, sulfate adenylyltransferase, adenylyl-sulfate kinase, phosphoadenosine phosphosulfate (PAPS) reductase, and sulfite reductase using BLASTp analysis: *CYS13, CYS14, CYS11, ADSK1, CYS5, CYS2*, and *CYS4* (Table 1). When we analyzed the transcript levels of these genes under sulfur-deprived conditions, all genes were upregulated in the wild-type strain 2 h after being transferred to a sulfur-deficient condition (Fig. 3). In particular, the putative sulfate permease genes *CYS14* and the putative PAPS reductase genes *CYS5* were remarkably induced by 1594 and 372 times, respectively. Other genes were also significantly upregulated (>13-fold) under sulfur depletion conditions in the wild type. However, in the *FgbZIP007* deletion mutant, the transcript levels of these genes decreased or exhibited no significant change when transferred to a sulfur depletion condition. These results show that Fgbzip007 positively regulates the expression of genes involved in the sulfur assimilation pathway in response to sulfur starvation.

**TABLE 1:** Identification of sulfur assimilation pathway genes in *Fusarium graminearum*

Gene name	Annotation	Locus ID	Number of amino acids	*Neurospora crassa*		*Aspergillus nidulans*	
				Gene name	Identity (%)	Gene name	Identity (%)
*CYS13*	Family sulfate permease	FGSG_01066	812	*cys-13*	54	*sB*	52
*CYS14*	Family sulfate permease	FGSG_02163	786	*cys-14*	49	*sB*	58
*CYS11 (MET3*)	Sulfate adenylyltransferase	FGSG_08875	574	*cys-11*	78	*sC*	74
*ADSK1*	Adenylyl-sulfate kinase	FGSG_01329	207	*adsk-1*	78	*sD*	67
*CYS5*	Phosphoadenosine phosphosulfate reductase	FGSG_08528	316	*cys-5*	49	*sA*	55
*CYS4*	Sulfite reductase subunit beta	FGSG_02482	1,535	*cys-4*	75	*sF* (AN7600)	66
*CYS2*	Sulfite reductase (NADPH) flavo alpha-component	FGSG_08463	1,065	*cys-2*	66	AN1752	55

To investigate the role of Fgbzip007 in sulfur source utilization, we observed the growth of the wild-type, Δ*fgbzip007*, and *FgbZIP007*c strains on modified minimal media (MMM) supplemented with various inorganic and organic sulfur sources (Fig. 3B). The Δ*fgbzip007* mutant was inviable on MMM in the presence and absence of inorganic sulfur sources. Also, glutathione, an organic sulfur source, was unable to rescue the growth defects of Δ*fgbzip007*. The growth impairment of Δ*fgbzip007* was entirely restored in the presence of methionine and homocysteine but it was only partially restored when cysteine was added. These results suggest that Fgbzip007 plays an important role in the utilization of inorganic sulfur sources by which Fgbzip007 regulates the expression of genes involved in sulfur assimilation.

### Fgbzip007-mediated sulfur metabolism is required for glutathione biosynthesis

**Fig 4:**
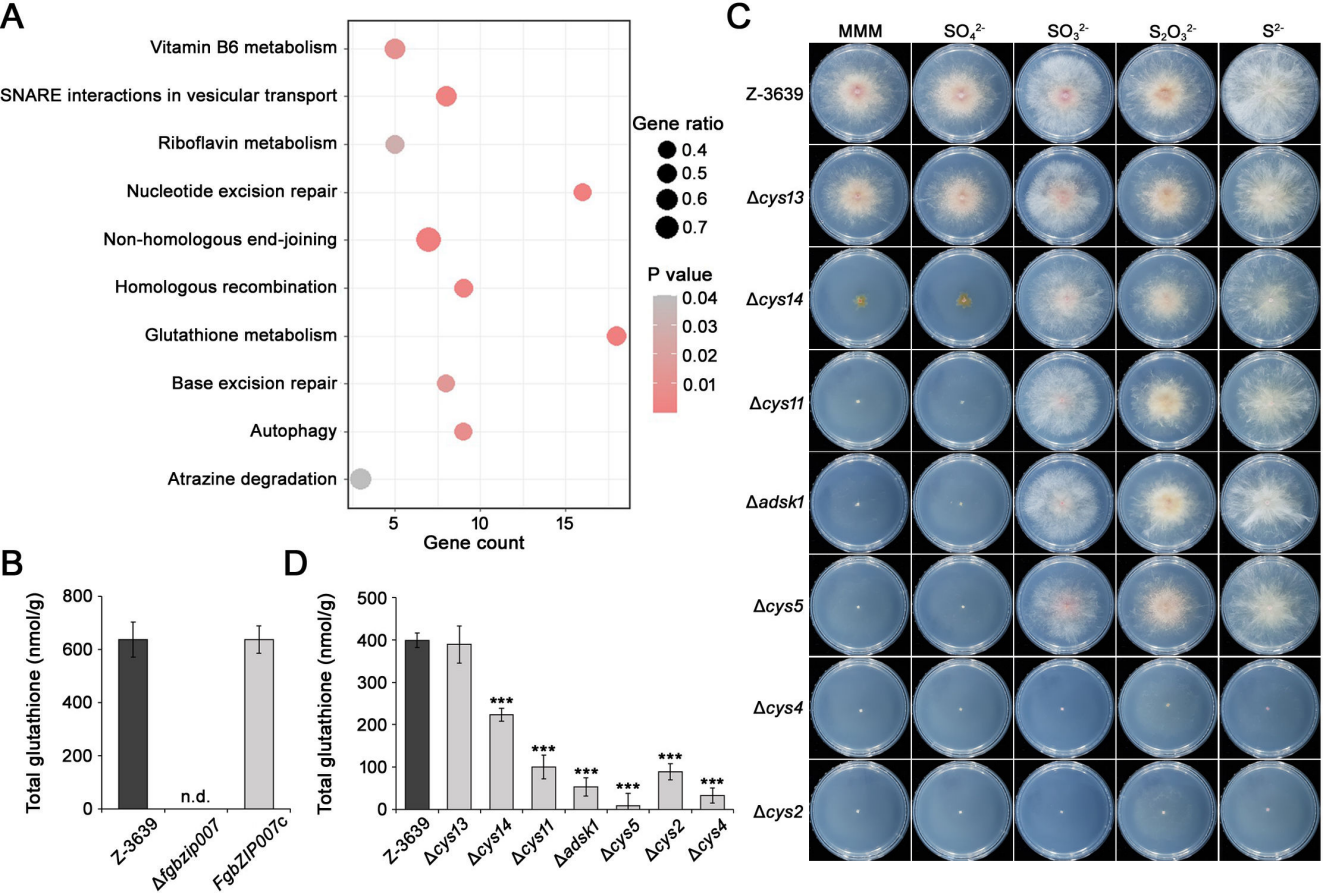
Glutathione deficiency in sulfur assimilation-deficient mutants. (**A**) Scatterplot for the top 10 enriched KEGG pathways of upregulated DEGs. The y-axis represents the enriched pathway, and the x-axis represents the number of genes enriched in the pathway. The dot sizes indicate the rich factor. (**B**) Quantification of glutathione in the wild type and Δ*fgbzip007* mutant. n.d., not detected. (**C**) Vegetative growth of the wild type and sulfur assimilation mutants on modified minimal media (MMM) with various inorganic sulfur sources. Photographs were captured 4 days after inoculation. (**D**) Quantification of glutathione in the sulfur assimilation-deficient mutants. Error bars indicate the standard deviation of the means (***P < 0.001; *t*-test).

Considering that Fgbzip007 is a key regulator of the sulfur assimilation pathway and is involved in hypersensitivity to oxidative stress, we investigated the association between sulfur metabolism and the oxidative stress response. KEGG enrichment analysis using our previous RNA-seq results derived from Δ*fgbzip007* and H_2_O_2_-treated Δ*fgbzip007* (27) revealed that differentially expressed genes (DEGs) were mainly enriched in “non-homologous end-joining (fgr03450),” “atrazine degradation (fgr00791),” “vitamin B6 metabolism (fgr00750),” “SNARE interactions in vesicular transport (fgr04130),” and “glutathione metabolism (fgr04480)” (Fig. 4A). Among them, we focused on the biosynthetic pathway for glutathione that is a representative antioxidant compound comprised of cysteine, glutamic acid, and glycine (28, 29). Cysteine is synthesized *via* the sulfur assimilation pathway (30, 31). Therefore, we hypothesized that the deletion of *FgbZIP007* would have an impact on glutathione biosynthesis. When we measured glutathione levels in each fungal strain, there was no detectable level of glutathione in the Δ*fgbzip007* mutant, whereas the levels of glutathione were restored to wild-type levels in the *FgbZIP007*-complemented stain (Fig. 4B).

To further dissect the mechanisms through which the sulfur assimilation pathway mediated by Fgbzip007 is involved in glutathione biosynthesis, we generated targeted-gene knockout mutants of the sulfur assimilation pathway (Table 1). To investigate sulfur source utilization in these mutants, all mutants were cultured on MMM and MMM supplemented with various inorganic sulfur sources (Fig. 4C). *CYS13* deletion did not affect on development under MMM. By contrast, Δ*cys14* exhibited severe growth defects in MMM and MMM containing SO_4_^2−^. The Δ*cys11*, Δ*adsk1*, and Δ*cys5* mutants were able to grow normally in the presence of SO_3_^2−^, S_2_O_3_^2−^, and S^2−^. The Δ*cys2* and Δ*cys4* mutants showed severe growth defects in MMM even when treated with inorganic sulfur sources. These results indicate that those genes are required for the utilization of inorganic sulfur sources.

We quantified the glutathione levels of sulfur assimilation pathway mutants and found that the glutathione contents of all mutants were significantly lower than that of the wild type, except for Δ*cys13* strain (Fig. 4D). In particular, glutathione levels in knock-out mutants of the genes encoding Cys11, Adsk1, Cys5, Cys2, and Cys4, which were identified as direct binding targets of Fgbzip007, were reduced to less than 25% of those in the wild-type strain. Therefore, our results suggest that Fgbzip007-mediated sulfur assimilation is required for glutathione biosynthesis in *F. graminearum*.

### Glutathione metabolism is required for oxidative stress resistance in *F. graminearum*

**Fig 5:**
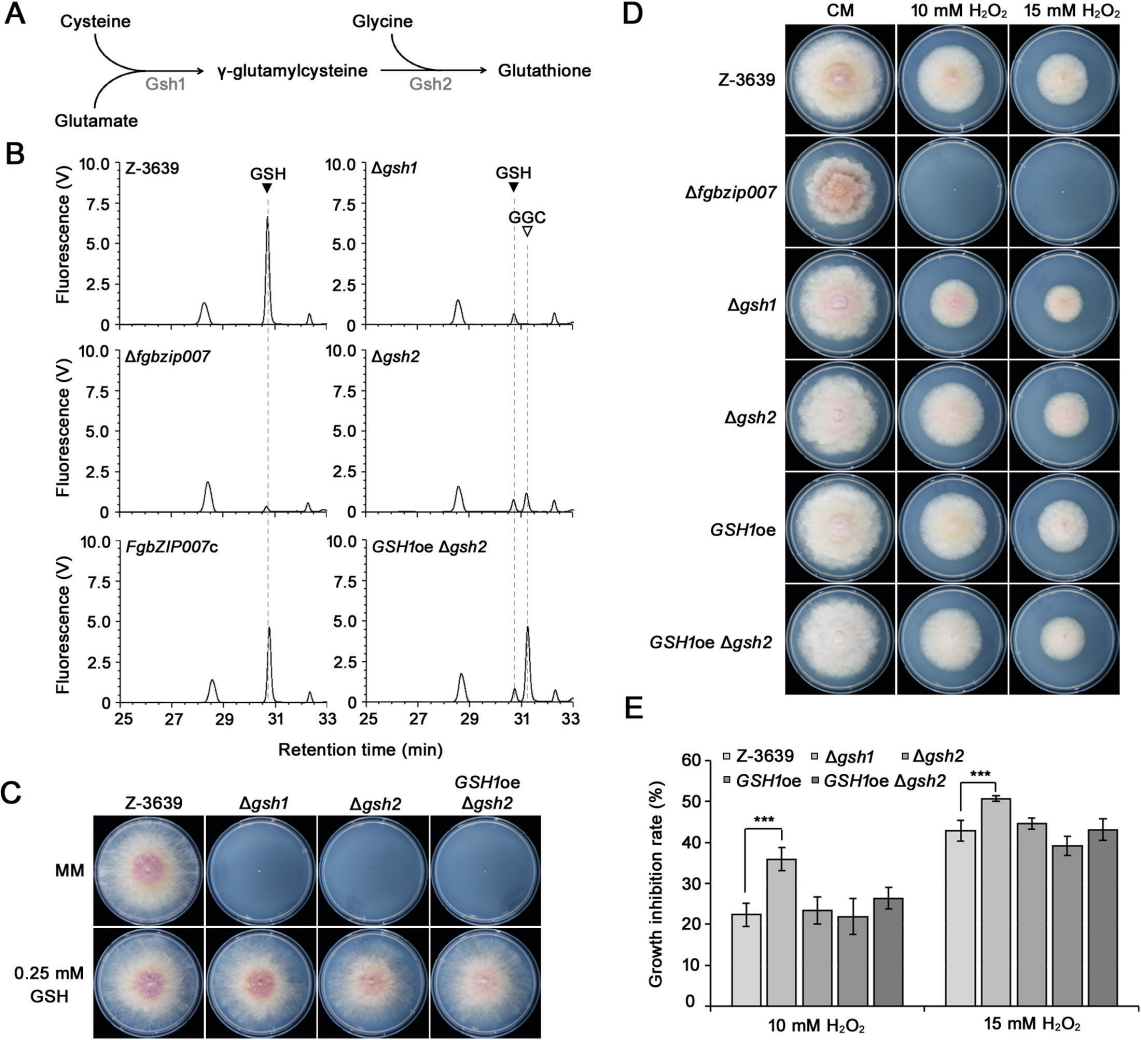
The role of glutathione metabolism in oxidative stress response. (**A**) Schematic representing glutathione biosynthesis process. (**B**) HPLC profiling of glutathione and γ-glutamylcysteine extracted from the wild-type strain and glutathione biosynthesis mutants. GSH, glutathione; GGC, γ-glutamylcysteine. (**C**) Vegetative growth of glutathione biosynthesis mutants on minimal medium (MM) and MM supplemented with 0.25 mM glutathione. Pictures were taken 4 days after inoculation. (**D**) Oxidative stress sensitivity of glutathione biosynthesis mutants. Each strain was cultured on CM and CM was treated with 10 and 15 mM H_2_O_2_. Pictures were taken 5 days after inoculation. (**E**) Statistical analysis of mycelial growth inhibition under oxidative stress conditions. Error bars indicate the standard deviation (***P < 0.001; *t*-test).

To determine whether the hypersensitivity of Δ*fgbzip007* to oxidative stress is caused by a glutathione deficiency, we identified genes involved in the glutathione biosynthesis pathway in *F. graminearum*. Glutathione is synthesized by two enzymatic steps. First, glutamic acid and cysteine are catalyzed by γ-glutamylcysteine synthetase. Glycine is then conjugated to γ-glutamylcysteine by glutathione synthetase. We designated the two genes encoding γ-glutamylcysteine synthetase and glutathione synthetase as *GSH1* and *GSH2*, respectively (Fig. 5A). We constructed glutathione depletion mutants by deleting *GSH1* (Δ*gsh1*) and *GSH2* (Δ*gsh2*). In addition, Δ*gsh2* mutants with *GSH1* overexpression (*GSH1*oe Δ*gsh2*) were generated to confirm the function of the intermediate, γ-glutamylcysteine, in the absence of glutathione.

Using high-performance liquid chromatography (HPLC) analysis, we then confirmed the production of glutathione and γ-glutamylcysteine in each strain (Fig. 5B). At a retention time of 30.25 min, a specific peak of glutathione was observed in both the wild-type and *FgbZIP007*-complemented strains. By contrast, the glutathione peak area in the Δ*fgbzip007* mutant was drastically reduced to about 6% of the wild-type strain, which is consistent with the result of Fig. 4B. In addition, the Δ*gsh1* and Δ*gsh2* deletion mutants exhibited decreased peak areas corresponding to the glutathione standard. Compared to the wild-type strain and Δ*gsh1* mutants, the Δ*gsh2* showed an additional peak corresponding to the standard γ-glutamylcysteine. In *GSH1*oe Δ*gsh2*, the peak area corresponding to the γ-glutamylcysteine was dramatically increased. We additionally obtained *GSH1*-overexpressing strains (*GSH1*oe) and confirmed that glutathione was overproduced (Fig. S2). These results suggest that *GSH1* and *GSH2* play a direct role in the glutathione biosynthesis pathway.

To determine the effect of glutathione deficiency on fungal growth, we compared the growth of each strain on MM in the absence and presence of glutathione (Fig. 5C). The Δ*gsh1* and Δ*gsh2* mutants were unable to grow on MM but their growth was restored in the presence of glutathione. These results suggest that glutathione is essential for the vegetative growth of *F. graminearum* and that the role of glutathione cannot be replaced by γ-glutamylcysteine.

To investigate the role of glutathione metabolism in oxidative stress resistance, each strain was cultured on CM supplemented with 10 and 15 mM hydrogen peroxide (Fig. 5D and E). Compared with the wild type, Δ*gsh1* was more susceptible to oxidative stress. By contrast, the Δ*gsh2* and *GSH1*oe Δ*gsh2* mutants displayed a similar tolerance to oxidative stress as the wild type, despite the depletion of glutathione in those strains. These results indicate that glutathione metabolism is required for oxidative stress resistance, and γ-glutamylcysteine itself can function as an antioxidant.

### Glutathione is essential for pathogenicity in *F. graminearum*

**Fig 6:**
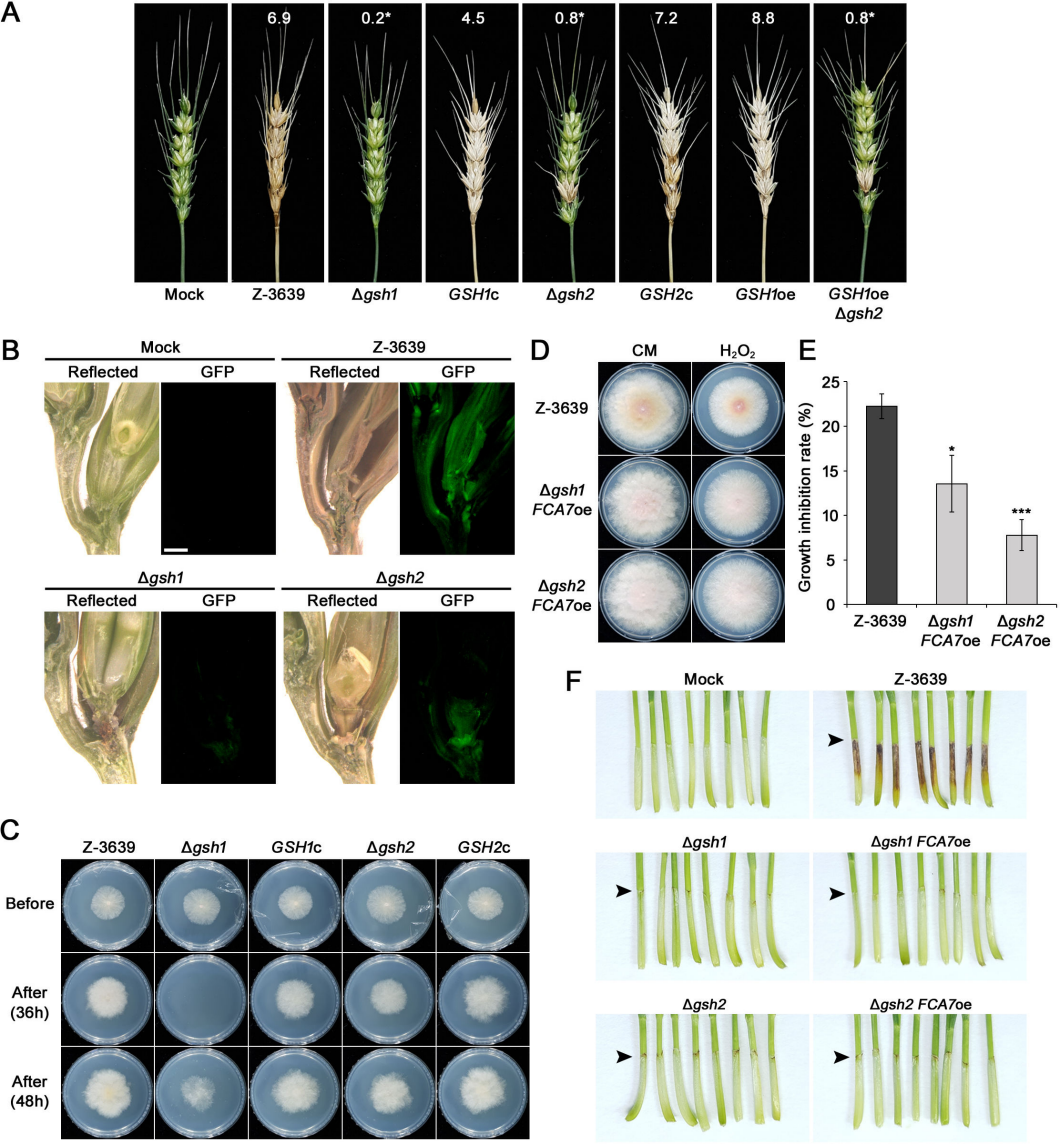
The role of glutathione in the pathogenicity of *F. graminearum*. (**A**) Virulence on wheat heads. The center spikelet of each wheat head was injected with 10 µL of a conidial suspension. Photographs were captured 21 days after inoculation. (**B**) Microscopic images of the cross-sections of the wheat spikes after inoculation with fungal strains. The conidial suspension of the strains expressing green fluorescent protein (GFP) in the cytosolic region was injected into the center spikelet. Infected wheat heads were harvested at 6 days after inoculation and were longitudinally sectioned. GFP fluorescence signal indicates fungal hyphal growth spreading from the inoculation sites. Arrowheads indicate the inoculated spikelets. “Reflected” means reflected light. Scale bar = 1,000 µm. (**C**) Cellophane membrane penetration by *F. graminearum* strains. Each strain was grown on complete media (CM) covered with a cellophane membrane for 36 and 48 h. The pictures were taken 48 h after removing the cellophane membrane. (**D**) Oxidative stress sensitivity of Δ*gsh1* and Δ*gsh2* mutants with *FCA7* overexpression. Photographs were taken 5 days after inoculation. (**E**) Statistical analysis of mycelial growth inhibition under oxidative stress conditions. Asterisks represent significant differences from the wild type (*P < 0.05; ***P < 0.001; *t*-test). (**F**) Representative pictures of wheat seedlings inoculated with *F. graminearum* strains. Coleoptiles of 2-day-old wheat seedlings were cut and inoculated with conidial suspensions of *F. graminearum* strains. The pictures were taken 7 days after inoculation. The arrows indicate the inoculation sites.

To determine the role of glutathione metabolism in pathogenicity, a virulence test was conducted on flowering wheat heads. While the wild-type strain caused typical symptoms of FHB, the mutants with defects in glutathione metabolism showed significantly reduced virulence (Fig. 6A). The spikelets inoculated with Δ*gsh1* mutant exhibited no or only marginal symptoms. The Δ*gsh2* and *GSH1*oe Δ*gsh2* mutants caused symptoms only on an inoculated spikelet. The complemented strains exhibited recovered virulence, causing normal FHB symptoms. To confirm hyphal growth during infection on wheat heads, we generated cytosolic GFP expression mutants by introducing the pIGPAPA vector into the wild-type, Δ*gsh1*, and Δ*gsh2* strains. The GFP signal of the wild type indicates that the mycelia had spread to adjacent spikelets through rachis nodes 6 days after inoculation (Fig. 6B). By contrast, the GFP signal of Δ*gsh1* was not observed, and the fluorescence signal of Δ*gsh2* was only observed in the inoculated spikelets.

Based on the results that the glutathione-deficient mutants caused the symptoms only restricted to the inoculated sites, we hypothesized that disruption of glutathione biosynthesis caused impairment in the penetration ability and conducted cellophane membrane penetration assays at different time points. Fungal strains were inoculated on the potato dextrose agar (PDA) media overlaid with the cellophane membrane. The cellophane membrane was removed after 36 h, and the resulting cultures were incubated for an additional 2 days. The Δ*gsh1* deletion mutant showed no growth on the media when the wild-type strain was able to penetrate the membrane. Interestingly, we observed the growth of the Δ*gsh2* mutant on PDA (Fig. 6C). Prolonged incubation for 48 h before removing the cellophane membrane resulted in all the fungal strains growing on PDA but the Δ*gsh1* mutant showed attenuated growth due to delayed penetration. These results suggest that, although the accumulation of γ-glutamylcysteine can partially compensate for the defects of glutathione-deficient strains in penetration ability, glutathione biosynthesis is essential for pathogenesis.

To explore whether the reduced virulence of glutathione-deficient mutants is restored by an enzymatic oxidant system such as *FCA7* encoding a putative bifunctional catalase-peroxidase, we further generated an overexpression strain of *FCA7* based on the glutathione-deficient mutants Δ*gsh1* and Δ*gsh2*, which were named Δ*gsh1 FCA7*oe and Δ*gsh2 FCA7*oe, respectively. These mutants displayed increased resistance to oxidative stress compared to the wild-type strain (Fig. 6D and E).

Although both Δ*gsh1 FCA7*oe and Δ*gsh2 FCA7*oe strains increased resistance to oxidative stress compared to the wild type, overexpression of *FCA7* did not restore the defects in virulence of Δ*gsh1* and Δ*gsh2* when these mutants were inoculated on the coleoptile (Fig. 6E and F); the Δ*gsh1* and Δ*gsh2* strains exhibited defective infection, causing dark discoloration only in the inoculated regions, which was consistent with the infection assay on wheat heads (Fig. 6A and F). Considering that the increased resistance to oxidative stress was unable to restore the pathogenicity of glutathione deficiency strains, our findings suggest that glutathione plays an essential role in full virulence independent of antioxidant systems.

## DISCUSSION

TFs have significant roles in diverse biological processes by orchestrating gene expression. Studies on TFs have been of particular interest in the Kingdom Fungi, given the presence of fungal-specific TFs (32, 33), and the goal of these studies was to unravel their regulatory mechanisms and identify targets for disease control. Among TF domains, the bZIP domain is one of the most abundant families found in eukaryotes, and bZIP proteins play roles in development, stress responses, and nutrient utilization (34–36). However, the downstream regulatory network is still unclear in most bZIP TFs. Although ChIP-seq is a powerful tool in that it can identify the direct TF regulon, ChIP-seq has rarely been performed because of technical obstacles associated with filamentous fungi. In this study, we successfully performed ChIP-seq analysis and identified the regulon of the bZIP protein, Fgbzip007. Fgbzip007 directly regulates the expression of enzymes involved in the sulfur assimilation pathway, consistent with the function of CYS3/METR reported in various fungi (37–40). Previous studies on *N. crassa*, *Penicillium expansum*, and *A. nidulans* have shown that the deletion of *cys-3*/*metR* significantly reduces the transcript levels of sulfur assimilation genes and their enzyme activities, aligning with the observations in this study (21, 37, 38). In addition, in *N. crassa*, random sequence oligonucleotides and DAP-seq analysis revealed CYS3 binding motifs as 5′-ATBRCGCCATC-3′ and 5′-ATGGCGCCAT-3′ (25, 41), which exhibit high similarity to the motif analysis results in this study. Overall, these findings demonstrate that Fgbzip007 is a functional ortholog of the CYS3/METR and, like typical bZIP proteins, plays a significant role in a wide range of mechanisms including nutrient utilization and stress response.

As a result of the sulfur assimilation process regulated by Fgbzip007 being involved in the biosynthesis of glutathione, an S-containing antioxidant, it has been confirmed that glutathione serves as another mechanism through which Fgbzip007 is involved in oxidative stress response, in addition to the regulation of Fca7 expression as confirmed in the previous study (20). The glutathione mechanisms that are accompanied by the actions of glutathione-dependent enzymes, including glutathione peroxidase (GPx) and reductase (GR), have been well studied. GPx and GR are involved in redox regulation by conversion between reduced glutathione (GSH) and oxidized glutathione (GSSG) (42), and it has been well investigated that these enzymatic antioxidant systems are required for oxidative stress resistance and pathogenicity in various plant pathogenic fungi such as *Magnaporthe oryzae*, *Alternaria alternata*, and *Valsa mali* (43–45). In this study, we diverged from the previous focus on enzymatic antioxidants and instead centered our investigation on the glutathione biosynthetic pathway. This allowed us to explore the non-enzymatic antioxidant function of glutathione itself. Here, we confirmed that the Δ*gsh1* deletion mutant, in which glutathione biosynthesis is completely collapsed, exhibited increased sensitivity to oxidative stress, verifying the role of glutathione in the oxidative stress response. Interestingly, the accumulation of γ-glutamylcysteine, an intermediate product in the glutathione biosynthesis process, was able to restore oxidative stress sensitivity caused by glutathione deficiency. This result suggests that γ-glutamylcysteine also functions in oxidative stress response. In *S. cerevisiae*, it was reported that the deletion of *GSH2* caused no significant difference in oxidative stress resistance (46), which is in agreement with our results. Based on reports of the reactivity of thiol-containing peptides or compounds with hydrogen peroxide or other peroxides (47, 48), it is considered that γ-glutamylcysteine can function as an *in vivo* scavenger of ROS.

Previous studies on the glutathione mechanism have linked its role in oxidative stress response to pathogenesis but this study showed that glutathione plays an essential role in pathogenicity independently of the resistance to oxidative stress. We observed that the accumulation of γ-glutamylcysteine and the overexpression of *FCA7* could not restore the pathogenicity of glutathione-deficient mutants. As the penetration ability of fungi is an important factor in their pathogenicity, we examined penetration in glutathione-deficient strains. Although we observed a retarded penetration in the Δ*gsh1* mutant, this was restored by the accumulation of γ-glutamylcysteine, confirming that penetration ability is not the primary cause of the impairment of pathogenicity shown in glutathione-deficient strains. Then, how does glutathione contribute to pathogenicity? First, we propose that glutathione may influence pathogenicity by participating in redox signaling. Glutathione itself can act as a signaling molecule by binding to thiol residues on target proteins, and this process is referred to as S-glutathionylation (49). S-glutathionylation-related mechanisms, including glutaredoxin, are involved in the regulation of transcription factors and kinase activity (50–52). Furthermore, it has been reported that the S-glutathionylation system is required for pathogenicity in various fungi, including *Cryptococcus neoformans* and *A. alternata* (53, 54). Second, glutathione is regarded as an essential nutrient for cell survival. As shown in this study, glutathione-deficient strains were unable to grow on MM, underscoring the indispensable role of glutathione in fungal growth. Moreover, *GSH1* has been reported as an essential gene in *S. cerevisiae*, *Candida glabrata*, *A. nidulans*, and *Aspergillus oryzae* (55–57). Given the vital role of glutathione in cell survival, it is possible that disruption of glutathione biosynthesis causes defects in pathogenicity. Hence, while glutathione serves as an antioxidant, it also functions in various intracellular processes, indicating its potential contribution to pathogenicity, and further research is needed to clarify its role in pathogenicity.

**Fig 7:**
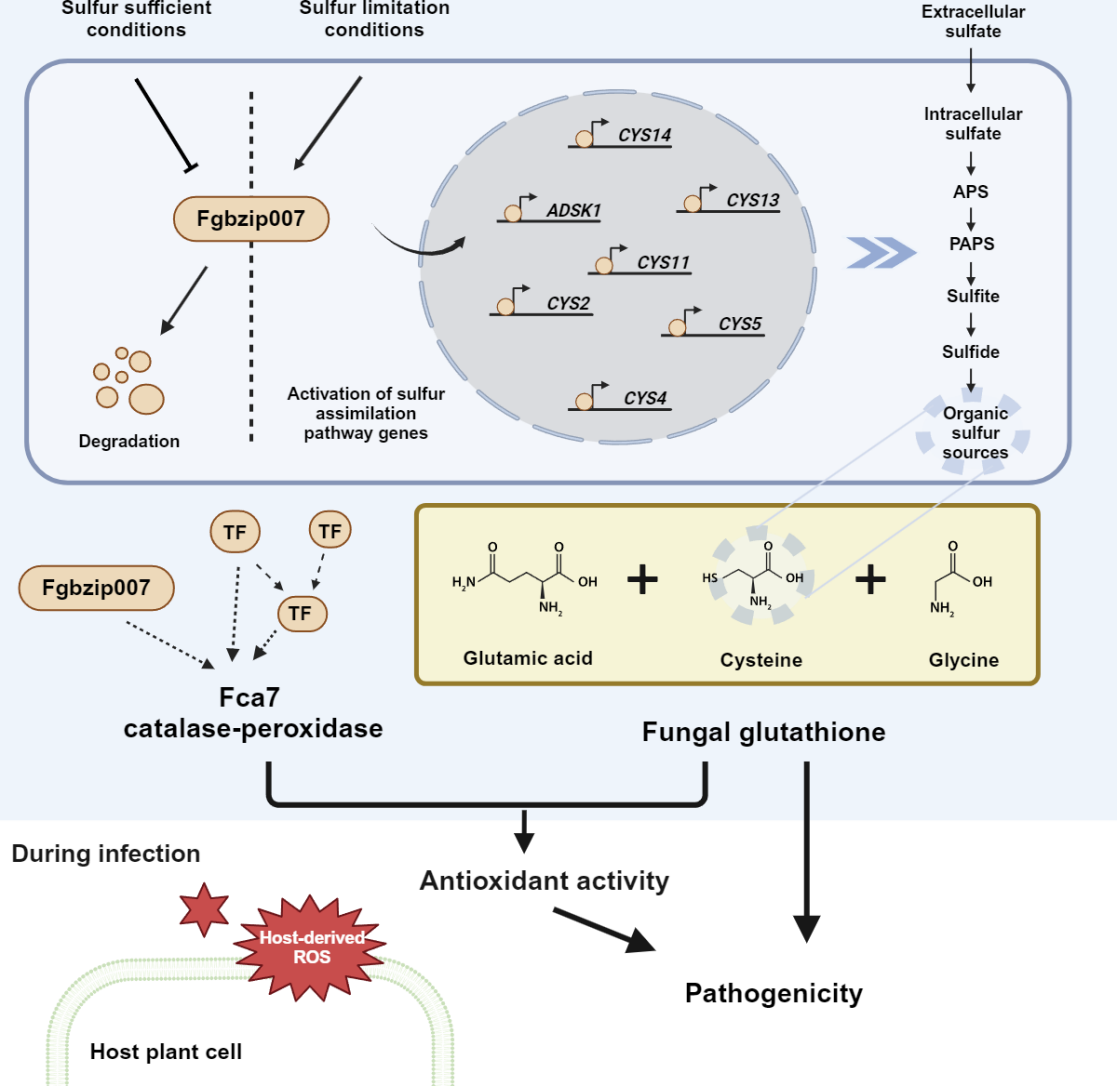
Proposed model for the mechanisms underlying Fgbzip007 on pathogenicity and oxidative stress response in *F. graminearum*. Fgbzip007 plays an important role in a variety of mechanisms and is crucial for toxicity. Under sulfur-deficient conditions, Fgbzip007 acts by binding to genes related to sulfur assimilation, thereby regulating their expression. Sulfur assimilation directly affects the synthesis of glutathione, an S-containing compound. Along with the antioxidant systems identified to be genetically regulated by Fgbzip007, glutathione serves as a non-enzymatic antioxidant and directly participates in protecting against oxidative stress. Furthermore, glutathione also plays a vital role in pathogenicity through mechanisms that are distinct from the oxidative stress response. A schematic model was created with Biorender.com.

Based on our previous and current study, we propose a comprehensive picture of the mechanisms by which Fgbzip007 is involved in pathogenicity, as shown in Fig. 7. In conclusion, Fgbzip007 functions as a key regulator in sulfur assimilation pathway, directly impacting the synthesis of glutathione. This non-enzymatic antioxidant glutathione, along with the previously identified enzymatic antioxidant Fca7, plays a direct role in oxidative stress response and independently contributes significantly to pathogenicity. Our study indicates the multifaceted involvement of Fgbzip007 in various downstream mechanisms and highlights its functional importance in pathogenesis.

## MATERIALS AND METHODS

### Fungal strains and culture condition

The *F. graminearum* wild-type strain Z-3639 (58) and mutants derived from this strain were used in this study (Table S1). All strains were stored as mycelia in a 20% glycerol solution at −80°C. The culture media were prepared according to the Fusarium laboratory manual (59). Fungal cells were cultured in carboxymethylcellulose medium (CMC) and yeast malt agar (YMA) for conidiation assay and conidial morphology observation. MMM was used for growth tests on various sulfur sources, as previously described (38). To remove sulfur from the MM, MgSO_4_ was replaced with MgCl_2_, and all sulfate salts in the trace element solution were substituted with the corresponding chloride salts. MMM supplemented with 5 mM methionine was used as a sulfur-sufficient condition, and MMM containing 0.25 mM methionine was used as a sulfur-depleted condition (23). For testing the utilization of sulfur sources, 2 mM MgSO_4,_ Na_2_SO_3_, Na_2_S_2_O_3_·5H_2_O, and Na_2_S were added to MMM as individual inorganic sulfur sources. Organic sulfur sources included 1 mM methionine, cysteine, homocysteine, and 0.25 mM glutathione.

### Nucleic acid manipulation and genetic modification

Fungal genomic DNA was isolated from lyophilized mycelia as described previously (59). Restriction endonuclease digestion and agarose gel electrophoresis were performed following standard protocols (60). Southern blot hybridization was conducted according to the protocol from the North2South Chemiluminescent Hybridization and Detection Kit (Thermo Scientific, Waltham, MA, USA). An oligonucleotide probe was labeled according to the protocol of the North2South Biotin Random Prime DNA Labeling Kit (Thermo Scientific).

The double-joint (DJ) PCR strategy was used for target gene deletion (61). The 5′ and 3′ flanking regions were amplified from the genomic DNA of the wild-type strain with the primer pairs 5 F-5R/3 F-3R. A geneticin resistance cassette (*GEN*) was amplified from the plasmid pII99 (62). These fragments were fused by a second round of DJ PCR without adding any additional primers. Final PCR constructs were then amplified using nested primers to split the marker genes.

For the complementation of *fgbzip007* deletion mutants, the GFP-fusion construct was generated using the yeast gap repair approach (63). The open reading frame (ORF) and native promoter regions of *FgbZIP007* were amplified using the primer pair bZIP007 native-F and GFP-R. The amplified fragment and *Xho*I-digested pDL2 vector were co-transformed into the yeast strain PJ69-4A for homologous recombination, and transformants were selected for tryptophan prototrophy. The recombinant plasmid was isolated and retransferred into the *Escherichia coli* DH10B strain. All the correct clones were confirmed by PCR. The same approach was used for generating the other FLAG and GFP fusion constructs as described above. For the construction of the FLAG-tagged Fgbzip007 strain, the ORF region of *FgbZIP007* was amplified using the primer pair RP27-F and FLAG-R, and co-transformed into the yeast strain PJ69-4A with *Xho*I-digested pHZ126 vector (64). To generate *GSH1*-GFP and *GSH2*-GFP fusion constructs for complementation and overexpression, the ORFs of *GSH1* and *GSH2* were amplified using Native-F/GFP-R and RP27-F/GFP-R primer pairs, respectively, and transformed into the yeast strain PJ69-4A with *Xho*I-digested pDL2 vector (63). All generated plasmids were confirmed by sequencing.

Each fusion PCR product or the recombinant vector was transformed into the protoplast via PEG-mediated transformation, as previously described (65). Transformants were confirmed through Southern blot hybridization or qRT-PCR (Fig. S3). The primers used in this study were synthesized by an oligonucleotide synthesis facility (Bioneer, Daejeon, Republic of Korea) (Table S2), and DNA sequencing was performed by Macrogen Inc. (Seoul, Republic of Korea).

### Sexual reproduction assay

For induction of sexual reproduction, each strain was inoculated on carrot agar medium for 5 days. Aerial mycelia were gently removed from the media with 400 µL of 2.5% Tween 60 solutions, and the resulting media were incubated under near-UV light (wavelength: 352 nm; Sankyo Denki, Tokyo, Japan). Perithecia formation was observed after 7 days using a SteREO Lumar V12 (Carl Zeiss, Oberkochen, Germany).

### ChIP-seq analysis

For ChIP experiments, fungal mycelium was incubated in 50 mL of cross-linking buffer (0.4 M sucrose, 10 mM Tris-HCl, pH 8.0, 1 mM PMSF, and 1% formaldehyde) for 15 min, and the cross-linking was stopped by adding 2.6 mL 2 M glycine under shaking for 5 min. Mycelia pellets were collected by vacuum filtration and ground with liquid nitrogen. The powder was resuspended in 4 mL nuclei lysis buffer (250 mM HEPES, pH 7.5, 150 mM NaCl, 1 mM EDTA, 1% Triton X-100, 0.1% sodium deoxycholate, 10 mM DTT, adding a protease inhibitor cocktail) and incubated at 4°C for 1 h. The samples were divided into 8 aliquots of 500 µL and sonicated for 48 min. After centrifuging at 12,000 rpm for 10 min, the supernatant was collected, and immunoprecipitation was conducted using anti-FLAG magnetic beads (Sigma, M8823). Crosslinks were reversed by adding 5M NaCl, and DNA was precipitated after treatment with RNase A and proteinase K as previously described (66).

DNA sequencing was accomplished using the Illumina NovaSeq 6000 platform (Illumina, San Diego, CA, USA), and the DNA library was analyzed using a Galaxy web-based platform. Reads were trimmed and aligned to the *F. graminearum* genome sequence using Trim Galore (version 0.6.7) and Bowtie2 (version 2.5.0), respectively (67). Normalization was conducted using the bamCoverage (version 3.5.1.0.0) tool, and peaks were identified using MACS2 callpeak (version 2.2.7.1) (68–70). Sequence data from input DNA were used as a control. To identify the binding motif of Fgbzip007, we collected the peak regions from the genes commonly identified in all replicates and submitted them to MEME (version 5.4.1.) (71). All ChIP-seq data have been deposited in the NCBI Sequence Read Archive (SRA) under accession number PRJNA993579.

### ChIP-qPCR

The DNA sample was diluted 10-fold and used for qPCR. Primers were designed to be located in the peak region of the identified binding genes, *CYS2* and *CYS5*. The enrichment level was determined using the 2^−ΔΔCT^ method (72), and the cyclophilin gene (*CYP1*; FGSG_07439) was used as an internal control. The experiment was performed with three replicates, and primers are listed in Table S2.

### qRT-PCR

Total RNA was extracted from mycelia using an Easy-Spin Total RNA Extraction Kit (iNtRON Biotechnology, Seoul, Republic of Korea). The first-strand cDNA was synthesized using the SuperScript III First-Strand Synthesis System (Invitrogen, Carlsbad, CA, USA). qRT-PCR was performed using iTaq SYBR Green Master Mix (Bio-Rad, Hercules, CA, USA) and a CFX Real-Time PCR System (Bio-Rad). For normalization, the endogenous housekeeping gene cyclophilin gene (*CYP1*; FGSG_07439) was used as an internal control, and the primer sets used are listed in Table S2.

### Western blot analysis

For total protein extraction, fresh mycelia were ground in liquid nitrogen and re-suspended in 1 mL extraction buffer containing protease inhibitors. Lysates were sonicated and centrifuged at 13,000 rpm for 20 min. The resulting supernatants were quantified with Pierce 660 nm Protein Assay Reagent (Thermo Scientific) and were used for western blot analysis. After boiling the sample, the denatured proteins were separated on 10% SDS polyacrylamide gels and transferred to nitrocellulose membranes using the Trans-Blot Turbo Transfer System (Bio-Rad). Fgbzip007-FLAG proteins were detected with an anti-FLAG M2-Peroxidase antibody (Sigma, A8592) following the procedure described in the manufacturer’s guide and photographed using a Chemi-Doc imaging system (Bio-Rad).

### EMSA

EMSA was performed with the purified His-tagged Fgbzip007 protein. For the expression of Fgbzip007, the full-length cDNA of Fgbzip007 was amplified using the primer pair bZIP007 pET28 F/R. The PCR products and pET28α vector were digested with *Hind*III and *Nco*I restriction enzymes, and after purification, both resulting products were ligated with T4 DNA ligase at 16°C overnight. The constructed plasmid was transformed into *E. coli* DH10B strain, and confirmed by DNA sequencing. The recombinant plasmid was introduced into *E. coli* BL21-CodonPlus (DE3)-RIL competent cells. For induction of protein expression, isopropyl β-D-1-thiogalactopyranoside (IPTG) was added to a final concentration of 0.3 mM in LB medium, and the cultures were further incubated at 15°C for 16 h. Proteins were purified with His Mag Sepharose Excel (Cytiva, Marlborough, MA, USA) following the manufacturer’s protocol. Double-stranded DNA probes were labeled with the Biotin 3′ End DNA Labeling Kit (Thermo Scientific), and binding reactions were performed using the LightShift Chemiluminescent EMSA kit (Thermo Scientific) following the manufacturer’s recommendations. The unlabeled probes (cold probes) were used as competitors. The resulting membranes were detected by a Chemi-Doc imaging system (Bio-Rad).

### Total glutathione quantification

For total glutathione analysis with a colorimetric assay, conidial suspensions (1 × 10^6^ conidia/mL) were inoculated into liquid CM and cultured at 25°C for 18 h with shaking at 150 rpm. The fungal mycelia were harvested by vacuum filtration, washed three times with sterile water, and ground in liquid nitrogen. The mycelia powder (0.6 g) was sonicated for 2 min and centrifuged at 12,000 rpm for 10 min. The supernatants were used for glutathione quantification. The glutathione level of each sample was determined using the OxiSelect total glutathione (GSSG/GSH) assay kit (Cell Biolabs, San Diego, CA, USA) following the manufacturer’s protocol. These experiments were repeated three times with two biological replicates.

The HPLC analysis for quantification of glutathione and γ-glutamylcysteine was conducted following a protocol previously described with some modifications (73). Conidial suspensions (1 × 10^6^ conidia/mL) were inoculated in liquid CM. After incubation at 25°C for 18 h with shaking at 150 rpm, the mycelia were harvested, washed three times with sterile water, and freeze-dried. The lyophilized mycelia samples (0.1 g) were suspended in 4 mL 0.1 M HCl and were centrifuged at 12,000 rpm for 20 min. For reduction, 120 µL of the supernatant was mixed with 180 µL CHES buffer and 30 µL DTT, and the resulting samples were incubated for 1 h at room temperature (RT). The derivatization was then carried out by adding 20 µL of 15 mM monobromobimane (mBBr), and the mixture was protected from light for 15 min at RT. The reaction was stopped by adding 250 µL of 0.25% methanesulfonic acid.

The derivatized samples were analyzed using a Prominence LC-20AR HPLC system equipped with an RF-20A fluorescence detector (Shimadzu, Kyoto, Japan). The mobile phases A and B were 10% and 90% acidic methanol (adjusted pH to 3.9 with trifluoroacetic acid), respectively. An Agilent Pursuit XRs C18 column was used, and the flow rate was kept at 1.0 mL/min. The elution was performed as follows: 0–21 min, a linear gradient from 5 to 15% B; 21–33 min, a linear gradient from 15 to 100% B; and an isocratic elution in 100% B for an additional 5 min. Fluorescence was detected at excitation and emission wavelengths of 380 nm and 470 nm, respectively.

### Pathogenicity assay

To test the virulence of each strain on the wheat head, the susceptible wheat cultivar, Eunpamil, was used as previously described (33). Briefly, a 10 µL of conidial suspension (1 × 10^5^ conidia/mL) was injected into the central spikelet of wheat heads with 10 replicates. Infected wheat heads were covered with plastic bags for 3 days to maintain humidity. Disease symptoms were observed 21 days after inoculation.

For the coleoptile virulence test, the top 2 mm of the 3-day-old seedlings were cut off and inoculated with 2 µL of conidial suspensions (1 × 10^6^ conidia/mL). The treated seedlings were incubated in a moisture chamber, and disease symptoms were determined at 7 days post-inoculation (74).

### Cellophane membrane penetration assay

To examine the penetration ability, each strain was inoculated on cellophane-overlaid PDA. Cellophane membranes were removed after 36 and 48 h post-inoculation, respectively, and the resulting media were observed after removal. All penetration experiments were conducted three times.

## SUPPLEMENTAL MATERIAL

Supplemental material mbio.02401-23-s0001.docx Fig. S1-S3 and Tables S1 and S2.

ASM does not own the copyrights to Supplemental Material that may be linked to, or accessed through, an article. The authors have granted ASM a non-exclusive, world-wide license to publish the Supplemental Material files. Please contact the corresponding author directly for reuse.
