# Supplementary material for: Sulfur metabolism-mediated fungal glutathione biosynthesis is essential for oxidative stress resistance and pathogenicity in the plant pathogenic fungus Fusarium graminearum
Source: mBio. 2023 Dec 19;15(1):e02401-23. doi: 10.1128/mbio.02401-23 (PMC10790779; doi:10.1128/mbio.02401-23)
Supplement: Supplemental material — Fig. S1-S3 and Tables S1 and S2. [file mbio.02401-23-s0001.docx]

**Supplemental material**

**Fgbzip007-mediated sulfur metabolism is required for glutathione biosynthesis, which is crucial for pathogenicity**

Jiyeun Park^1^, Jae Woo Han^2^, Nahyun Lee^1^, Sieun Kim^1^, Soyoung Choi^1^, Hyun-Hee Lee^3^, Jung-Eun Kim^4^, Young-Su Seo^3^, Gyung Ja Choi^2,5^, Yin-Won Lee^1^, Hun Kim^2,5^* and Hokyoung Son^1,6^*

^1^Department of Agricultural Biotechnology, Seoul National University, Seoul, 08826, Republic of Korea

^2^Center for Eco-Friendly New Materials, Korea Research Institute of Chemical Technology, Daejeon, 34114, Republic of Korea

^3^Department of Integrated Biological Science, Pusan National University, Busan, 46247, Republic of Korea

^4^Research Institute of Climate Change and Agriculture, National Institute of Horticultural and Herbal Science, Jeju, 63240, Republic of Korea

^5^Department of Medicinal Chemistry and Pharmacology, University of Science and Technology, Daejeon, 34113, Republic of Korea

^6^Research Institute of Agriculture and Life Sciences, Seoul National University, Seoul, 08826, Republic of Korea

Address correspondence to Hokyoung Son, hogongi7@snu.ac.kr, or Hun Kim, hunkim@krict.re.kr


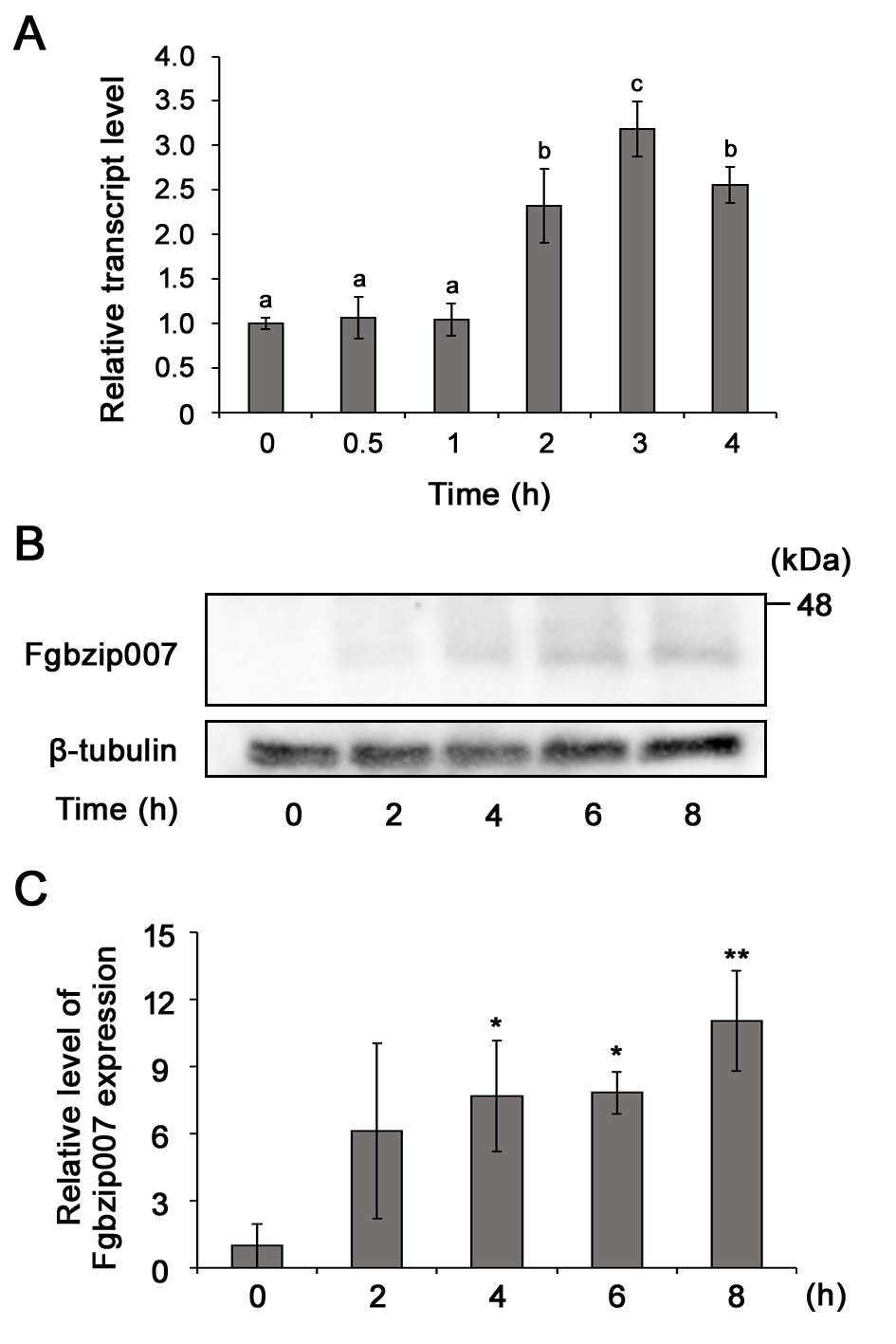


**Fig S1. Expression profile of Fgbzip007 under sulfur limitation condition.** (A) Relative transcript levels of *FgbZIP007* under sulfur limitation condition. The wild-type strain sample was prepared as described above, and total RNA was extracted from each time point after resuspension. Different letters on the bar indicate a statistically significant difference (p < 0.05; t-test). (B) Western blot analysis of the Fgbzip007 protein. Fgbzip007-Flag strain was grown in modified minimal media (MMM) with 5 mM methionine for 24 h. After washing with water, the resulting mycelia were transferred into MM with 0.25 mM methionine. Mycelia samples were harvested at different time points (0, 0,5, 1, 2, 3 and 4 h) and total proteins were extracted. Western blot assay was performed with an anti-FLAG antibody, and β-tubulin was used as an internal control. (C) The quantification of Fgbzip007 expression under sulfur limitation condition. The relative levels of protein expression were analyzed using three independent blots. Asterisks represent significant differences from the sample harvested at 0 hours (**P* < 0.05; ***P* < 0.01; *t*-test)


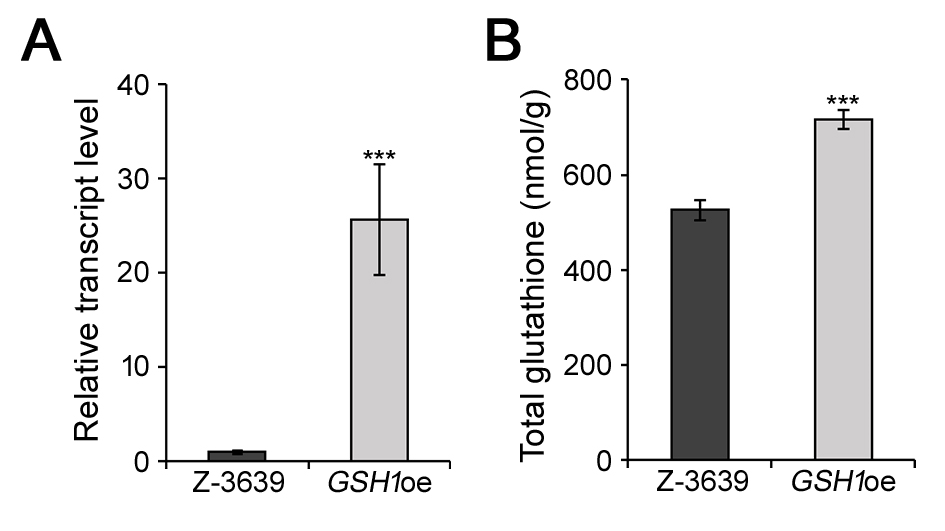


**Fig S2. Confirmation of the glutathione overproduction in *GSH1*oe strain.** (A) Relative transcript levels of *GSH1* in the wild-type and *GSH1*oe strains. The transcript levels of the gene were analyzed by quantitative real-time PCR amplification (qRT-PCR), and *CYP1* was used as a housekeeping gene. (B) Quantification of glutathione in the wild type and GSH1oe. Error bars indicate the standard deviation of the means (****P* < 0.001; *t*-test)


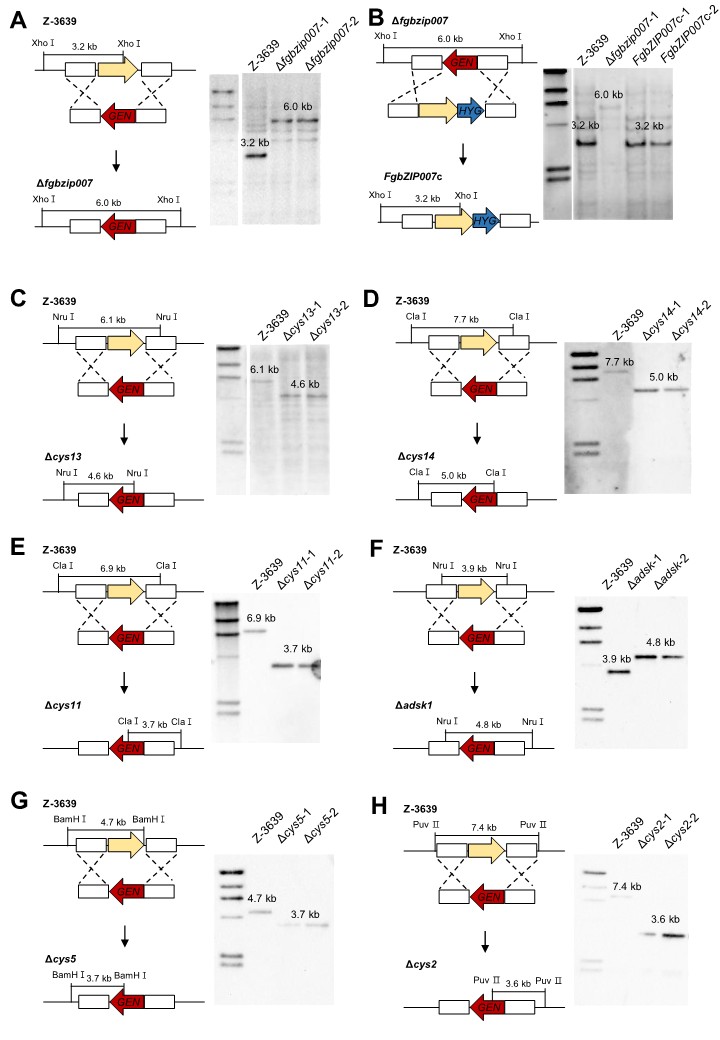

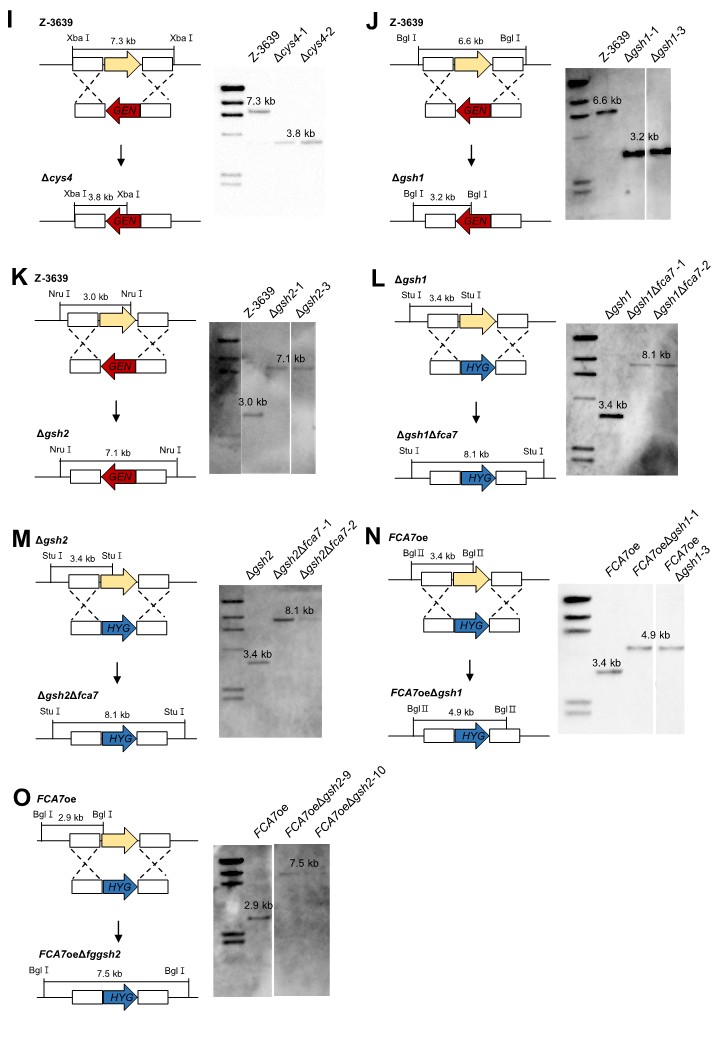


**Fig S3. Schematic representation of gene deletion strategy and Southern blotting of the deletion mutants used in this study.**

**Table S1. Strains used in this study.**

| Strain | Description/Comments | Reference |
| --- | --- | --- |
| Z-3639 | *F. graminearum* wild-type | Bowden and Leslie (1999) |
| Δ*fgbzip007* | Δ*fgbzip007::GEN* | Son et al. (2013) |
| *FgbZIP007*c | Δ*fgbzip007::FgbZIP007-GFP-HYG* | This study |
| Fgbzip007-FLAG | Δ*fgbzip007::FgbZIP007-GFP-HYG* | This study |
| Δ*cys13* | Δ*cys13::GEN* | This study |
| Δ*cys14* | Δ*cys14::GEN* | This study |
| Δ*cys11* | Δ*cys11::GEN* | This study |
| Δ*adsk1* | Δ*adsk1::GEN* | This study |
| Δ*cys5* | Δ*cys5::GEN* | This study |
| Δ*cys2* | Δ*cys2::GEN* | This study |
| Δ*cys4* | Δ*cys4::GEN* | This study |
| Δ*gsh1* | Δ*gsh1::GEN* | This study |
| *GSH1*c | Δ*gsh1::GSH1-GFP-HYG* | This study |
| *GSH1*oe | Δ*gsh1::RP27:GSH1:GFP:HYG* | This study |
| Δ*gsh2* | Δ*gsh2::GEN* | This study |
| *GSH2*c | Δ*gsh2::GSH2:GFP:HYG* | This study |
| *GSH1*oe Δ*gsh2* | Δ*gsh2::RP27:GSH1:GFP:HYG* | This study |
| HK12 | GFP*-HYG* (GFP constitutive expresser in cytosol) | Son et al. (2011) |
| Δ*gsh1*;pIGPAPA | Δ*gsh1::GEN,GFP-HYG* | This study |
| Δ*gsh2*;pIGPAPA | Δ*gsh2::GEN, GFP-HYG* | This study |
| *FCA7*oe | *FCA7::GEN-P_EF1α_-FCA7* | Lee et al. (2014) |
| Δ*gsh1 FCA7*oe | Δ*gsh1::HYG FCA7::GEN-P_EF1α_-FCA7* | This study |
| Δ*gsh2* *FCA7*oe | Δ*gsh2::HYG FCA7::GEN-P_EF1α_-FCA7* | This study |

**Table S2. Primers used in this study.**

| Primer name | Sequence (5’→3’) |
| --- | --- |
| bZIP007 5F | GCCGCATAGCAGCCAGTGTC |
| bZIP007 5R | gcacaggtacacttgtttagagCGATTGACGCCGAAGCCACTA |
| bZIP007 3F | ccttcaatatcatcttctgtcgATCTAGTGGGGACTATGTGGCGTTA |
| bZIP007 3R | GGTCATTGTGGTGCCTGGTATCTTC |
| bZIP007 5N | AGCGCGTCGTCTGATGTGTGA |
| bZIP007 5R | TGCCATCACACGTCTCACCAATAC |
| bZIP007 native/F | TATAGGGCGAATTGGGTACTCAAATTGGTTCTATGTAGTTCGGCAGATTTTGAGG |
| bZIP007 GFP/R | CCCGGTGAACAGCTCCTCGCCCTTGCTCACCCTTGTCTCATCCTTGACGGAG |
| bZIP007 RP27/F | TTTCGTAGGAACCCAATCTTCAAATCCATCAAAATGTCAAACTTCAACG |
| bZIP007 FLAG/R | TTTATAATCACCGTCATGGTCTTTGTAGTCCCTTGTCTCATCCTTGACGGAG |
| bZIP007 qRT-F | ATCAACCGTCAGGAGAATGCTCAC |
| bZIP007 qRT-R | TCATTCCATCCGAGGGAGAAGTG |
| CYS2 ChIP F | GCCCTTTTTTGTCATGCAACTTTC |
| CYS2 ChIP R | GACTCAACGGGACAACGGTAACAG |
| CYS4 ChIP F | AATCGAGGGCGAGGATTCATACA |
| CYS4 ChIP R | GCACGTCTTATTTTTCTCTTCCCACT |
| bZIP007 pET28 F | agatataccatgggcATGTCAAACTTCAACGGCCGC |
| bZIP007 pET28 R | ggccgcaagcttCCTTGTCTCATCCTTGACGGAGG |
| CYS2-EMSA-F | GTTCAATTGCGTGATTCTGTC |
| CYS2-EMSA-R | GACAGAATCACGCAATTGAAC |
| CYS5-EMSA-F | GCGTCGATGTCGTGATCAGTC |
| CYS5-EMSA-R | GACTGATCACGACATCGACGC |
| CYS13 qRT-F | ATGGGTGATCATTCAGTTCTCTCGTC |
| CYS13 qRT-R | TCCAATTCGAGAAGGGAAATAATGAA |
| CYS14 qRT-F | TCCTATCATGGGTGGGCAAATACA |
| CYS14 qRT-R | CAATGGTGATATCCTTGGATGTAGCA |
| CYS11 qRT-F | CAGAAGCTCCCTGCTCTTACACTCA |
| CYS11 qRT-R | GAGGTTGATGGGCATGGAGAAGAG |
| ADSK1 qRT-F | AGGTTGCTAAGCTGTTTGCCGATT |
| ADSK1 qRT-R | ACGTAGACCTCGACGAAGGGGATA |
| CYS5 qRT-F | GAATGCGAGTCAGGCTATGTCTCTG |
| CYS5 qRT-R | AGTCGATTGGTACAGGTTAGGGAACA |
| CYS2 qRT-F | AGCAGGTTCCTCTTTCATCCATCTC |
| CYS2 qRT-R | GTCGAAGGTCTCGGGAGAGTAAGAG |
| CYS4 qRT-F | GAGTTCTCCTCTCACCTGAACCGATA |
| CYS4 qRT-R | AGGATGGTGGATGTGGTTGTGACT |
| CYS13 5F | GGCTTGGAGTTGTGTTGTCGTTAC |
| CYS13 5N | GGAATGCACGTAATTTGAGAGGTTG |
| CYS13 5R | gcacaggtacacttgtttagagTTGGTGCTTTGTTCGGTCTGTATT |
| CYS13 3F | ccttcaatatcatcttctgtcgCGATGAGATGTTGGTTGTTTTGTG |
| CYS13 3N | CCCGAGAGTGGTTCATTTGCTTAC |
| CYS13 3R | TCAGACTCCTTTGACGACTCTGAT |
| CYS14 5F | CTAGAATGCCTGCATATCTGTCGTGT |
| CYS14 5N | ATCTGTCCTTTCGCCCACTGTATT |
| CYS14 5R | gcacaggtacacttgtttagagAGGATGTGATTGTGTGGGAAGAAAG |
| CYS14 3F | ccttcaatatcatcttctgtcgTTGCGTCTTGTCTATACCCGATTTA |
| CYS14 3N | GTCGGCTGGATATCAAAGGAACAT |
| CYS14 3R | AAGACAAACCATGGAGCATAACCC |
| CYS11 5F | ATGGCGACTAAATGCAACACAATG |
| CYS11 5N | CTGTGTCGCATCAACGTCAGAGA |
| CYS11 5R | gcacaggtacacttgtttagagTGAAAGACAACGAAACAGGAACAGA |
| CYS11 3F | ccttcaatatcatcttctgtcgGCTAGCTACGCCTGCCTACTGAT |
| CYS11 3N | CAAGAAGGCGGTCAAGAATAGTGG |
| CYS11 3R | GGGAGAGGTTGTCGCTAAGTTCAC |
| ADSK1 5F | GACGGTTTGCTCGCTCTACACAG |
| ADSK1 5N | ACCCCGAACTTACTGAATCACGAC |
| ADSK1 5R | gcacaggtacacttgtttagagTCTGGAAGTAAACAATTGGGGAAAT |
| ADSK1 3F | ccttcaatatcatcttctgtcgTGATGATGATGATTGCGTTTTGC |
| ADSK1 3N | TCGCCCAGTACAAAATGGTCAAC |
| ADSK1 3R | CGGTCCAGTGCTAGCAACTTCAG |
| CYS5 5F | GGACGGTTTGATTGAGGATGAGAT |
| CYS5 5N | ATAAAGCACTGCAACACTGAGATGG |
| CYS5 5R | gcacaggtacacttgtttagagTTTGTTAATGCGTGGACTTGTTGA |
| CYS5 3F | ccttcaatatcatcttctgtcgCACTCGCAACCAGACAAAAACTTAT |
| CYS5 3N | TGTTTGCTTCTTGCTTGGCTTTTA |
| CYS5 3R | GACCTTAAGGACGACAACAGCACA |
| CYS2 5F | TGGGCGTGTATCTTGTTGTCAGAA |
| CYS2 5N | ATCAGGGGCGTGGCATAGTTATT |
| CYS2 5R | gcacaggtacacttgtttagagTCAACGGGACAACGGTAACAGTA |
| CYS2 3F | ccttcaatatcatcttctgtcgGCGTTTGAATACAACCGAACATTT |
| CYS2 3N | GGACTGGTCCGCTAAACAAAGTG |
| CYS2 3R | GGCTAAAGCGTCCGACTGTTATTC |
| CYS4 5F | ATGGGGAAGGTTGATATACGGAAA |
| CYS4 5N | GTTTACGAAGGATTTGTTTGGGTCA |
| CYS4 5R | gcacaggtacacttgtttagagTGGAGGAGGGGAGGATGTATAAGA |
| CYS4 3F | ccttcaatatcatcttctgtcgGATAAGCCTTTGCGTGAAACATCT |
| CYS4 3N | ACGACGACATAAAGATGGGTGACA |
| CYS4 3R | TACTCAACCCTCTCGTTGCGTCT |
| GSH1 5F | ACGGCATTGAATTGGAGTCTGGT |
| GSH1 5N | AACGGCTTTCGGTTACACGGAG |
| GSH1 5R | gcacaggtacacttgtttagagCGTTCCCAAAGCCCTGAGGTTA |
| GSH1 3F | ccttcaatatcatcttctgtcgGGATCGATGGCCAAGAAGTGATG |
| GSH1 3N | CCCGGCGGTTAAATGCTGATA |
| GSH1 3R | ATCAGCACACCGCAGTACATCTAATC |
| GSH2 5F | CTGTAACGCCGAGTTTGCTGAAC |
| GSH2 5N | AATCGCTGGAGCAAAGTAGGAAAA |
| GSH2 5R | gcacaggtacacttgtttagagCTTGCGTGCTTTGGACCTTTAC |
| GSH2 3F | ccttcaatatcatcttctgtcgGTAGATAGCAGGGGCTGTAACGAC |
| GSH2 3N | ACCTTTTCGTGGCGCCATAATA |
| GSH2 3R | CCTCAGCATTGTCCAGCGTATG |
| GSH1 Native/F | TATAGGGCGAATTGGGTACTCAAATTGGTTCCTTGTCCGATTTGATATTTTTCAG |
| GSH1 RP27/F | TTTCGTAGGAACCCAATCTTCAAACATCATGGGCCTCTTGTAAGTCTT |
| GSH1 GFP/R | CCCGGTGAACAGCTCCTCGCCCTTGCTCACCGATGCCATCACTTCTTGGCCATCGA |
| GSH2 Native/F | TATAGGGCGAATTGGGTACTCAAATTGGTTGGATGCGTTTGTAGCTCAAGACTT |
| GSH2 RP27/F | TTTCGTAGGAACCCAATCTTCAAACGACATCATGGCATCTCTTACAAG |
| GSH2 GFP/R | CCCGGTGAACAGCTCCTCGCCCTTGCTCACTACTAGAGTGCAGCTATCCATACACCC |
| GSH1 HYG 5R | CGACCGGGAACCAGTTAACAACGTTCCCAAAGCCCTGAGGTTA |
| GSH1 HYG 3F | CCTCCACTAGCTCCAGCCAAGCCGGATCGATGGCCAAGAAGTGATG |
| GSH2 HYG 5R | CGACCGGGAACCAGTTAACAACTTGCGTGCTTTGGACCTTTAC |
| GSH2 HYG 3F | CCTCCACTAGCTCCAGCCAAGCCGTAGATAGCAGGGGCTGTAACGAC |
